# Facilitated biological reduction of nitroaromatic compounds by reduced graphene oxide and the role of its surface characteristics

**DOI:** 10.1038/srep30082

**Published:** 2016-07-21

**Authors:** Lei Li, Qi Liu, Yi-Xuan Wang, Han-Qing Zhao, Chuan-Shu He, Hou-Yun Yang, Li Gong, Yang Mu, Han-Qing Yu

**Affiliations:** 1 CAS Key Laboratory of Urban Pollutant Conversion, Collaborative Innovation Centre of Suzhou Nano Science and Technology, Department of Chemistry, University of Science and Technology of China, Hefei, China; 2 Jiangsu Key Laboratory of Chemical Pollution Control and Resources Reuse, School of Environmental and Biological Engineering, Nanjing University of Science and Technology, Nanjing 210094, Jiangsu Province, China

## Abstract

How reduced graphene oxide (RGO) mediates the reductive transformation of nitroaromatic pollutants by mixed cultures and the role of its surface characteristics were evaluated in this study. Different electron donors were applied to investigate the interaction between RGO and anaerobic microbes. Moreover, the influence of the surface properties of RGO on biological nitroaromatic removal was further elucidated. The results show that RGO could achieve an approximate one-fold rate increase of nitrobenzene reduction by mixed culture with glucose as an electron donor. Selective elimination of oxygen moieties on the RGO surface, such as quinone groups, decreased the nitrobenzene transformation rate, whereas doping nitrogen into the RGO framework exhibited a positive effect. The study indicates that graphene-based carbon nanomaterials have the potential to accelerate the biological transformation of nitroaromatic compounds and that the functionalization of these carbon nanomaterials, especially through surface modification, would further enhance the conversion efficiency of contaminants.

Nitroaromatic compounds (NACs) are widely utilized as pesticides, explosives, and synthesis intermediates^1^,^2^ and contribute to environmental pollution. The electron-withdrawing effect of the nitro groups and the stability of the benzene ring lead to the recalcitrance of the NACs through oxidative degradation^3^,^4^. The easy facile reduction of NACs is a favorable option because of the enhanced biodegradability of the reductive products^5^,^6^,^7^. However, the anaerobic reductive process is usually slow, and it is valuable to promote the removal efficiency by developing various technologies.

The unique physical and chemical properties of carbon nanomaterials constantly inspire researchers to actively explore potential environmental applications, e.g., sorbents and pollution prevention strategies. Graphene, identified as a two-dimensional (2D) carbon nanomaterial, has attracted extensive attention in the last decade^8^,^9^. Although the development of graphene faces challenges (e.g., potential biological toxicity^10^), the large surface area, high electrical conductivity and catalytic activity of graphene^11^,^12^,^13^ make it a fascinating material for the prospect of accelerating the degradation of pollutants.

Due to the hydrophobicity and the large surface area, graphene can adsorb NACs via π–π electron donor–acceptor interactions and charge electrostatic and polar interactions^14^. The high electrical conductivity and the oxygen moieties on graphene provide the opportunity to accelerate electron transfer similarly to the enhanced elimination of azo dyes by activated carbon and carbon nanotubes^15^,^16^,^17^. More importantly, the extracellular electron transfer process has been shown to play a crucial role in the biological removal of different pollutants, not only in natural environments but also in engineered systems^18^,^19^. Recently, graphene and graphene oxide were reported to increase the rates of anaerobic degradation of recalcitrant pollutants^20^,^21^. The presence of graphene could increase the formation of bound extracellular polymeric substances, which might interact with secreted redox active species to accelerate nitrobenzene biotransformation^21^. Graphene oxide was proposed as an electron shuttle for the increased redox conversion of contaminants in biological systems^20^. Meanwhile, surface modification of carbon materials would also affect their performance. Both oxidized carbon fiber^22^ and hydrogen pretreated activated carbon^16^ have been found to be able to enhance the capacity for biological azo dye removal. Toral-Sánchez *et al.* probed the effect of surface chemistry of graphene oxide by oxidation and thermal treatment on iopromide reduction but in a pure chemical system^23^. Table S1 summarized the previous studies to investigate the influence of carbon materials and their surface modification on the reduction of recalcitrant contaminants. As a result, there was lack of sufficient information about the role of graphene as well as its surface characteristics especially through the modification by nitrogen doping on the biological reduction of nitroaromatic compounds with mixed cultures.

Therefore, this study aims to decipher the interaction of reduced graphene oxide (RGO) with nitroaromatic transformation by anaerobic mixed cultures. The specific objectives of this study include: (1) to elucidate how RGO affects electron transfer in the reduction of NACs by mixed anaerobic microorganisms; and (2) to clarify the effect of RGO surface characteristics, including oxygen moieties and nitrogen doping, on biological nitroaromatic conversion. Nitrobenzene was selected as the model NAC in this study because it is widely used and listed as a priority pollutant in many countries^24^,^25^.

## Results and Discussion

### Properties of RGO, thermally pretreated RGO (TPRGO) and nitrogen-doped graphene (NG)

**Figure 1:**
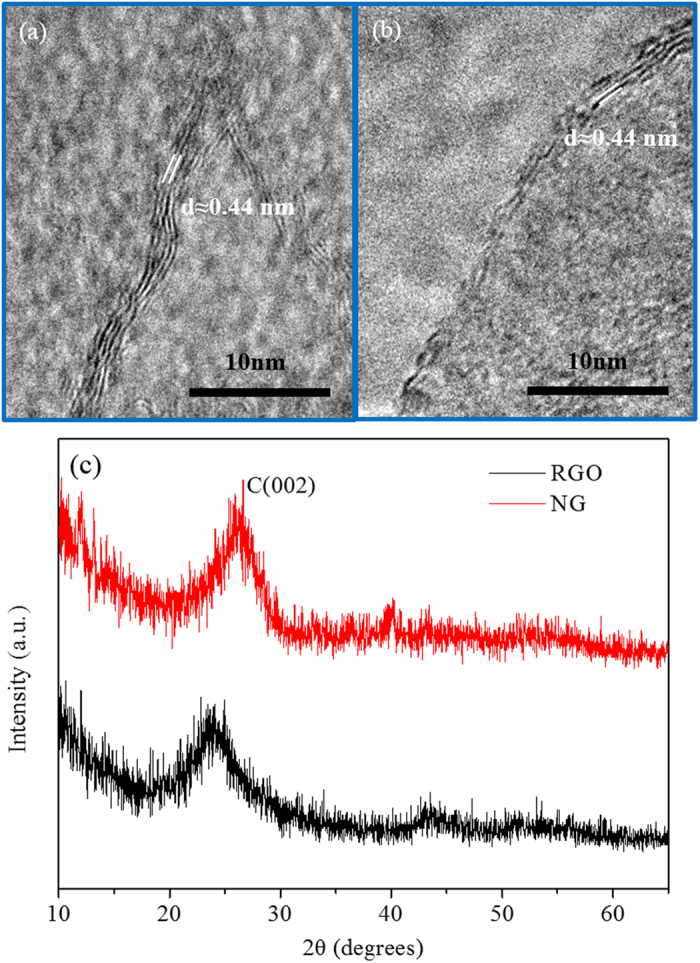
High-resolution transmission electron microscope (HRTEM) images of (**a**) reduced graphene oxide (RGO), (**b**) nitrogen-doped graphene (NG), and (**c**) X-ray diffraction (XRD) spectra of RGO and NG.

**Figure 2:**
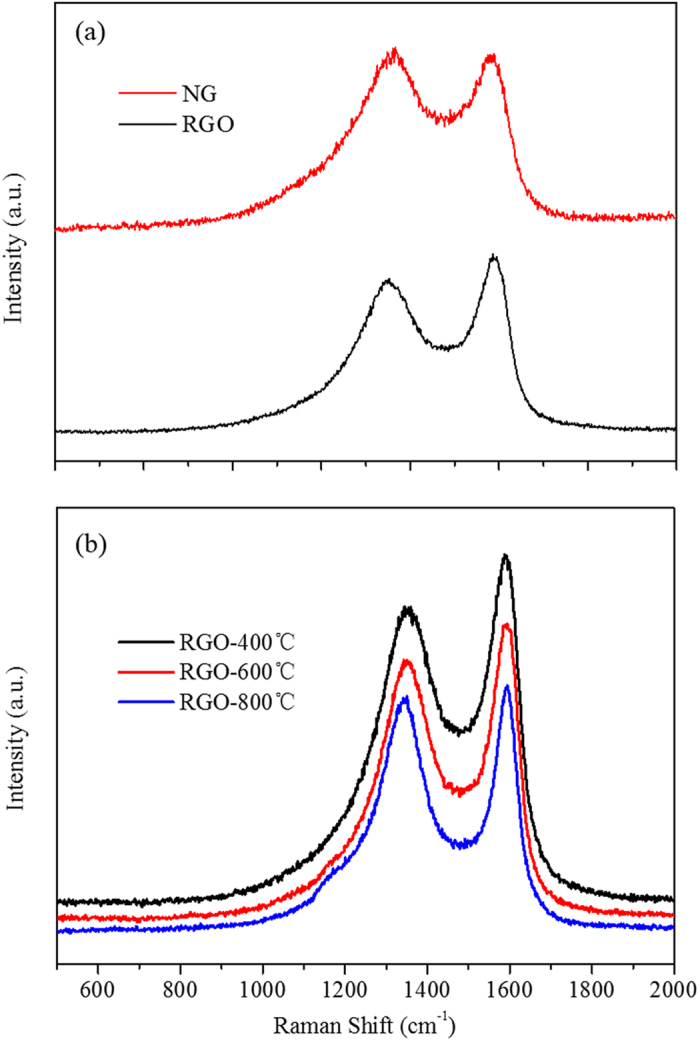
Raman spectra of (**a**) RGO and NG and (**b**) thermally treated reduced graphene oxide at 400 ^°^C, 600 ^°^C, and 800 ^°^C.

As shown in Fig. 1a,b, both RGO and NG had the microscopic 2 to 6 layers structure with similar interlayer distances. The (002) diffraction peak of RGO was found to be shifted to ~24° as compared to typical graphite (002) peak which is located at ~26.4° (Fig. 1c). The shift of (002) peak could be explained by the remaining oxygen moieties on the RGO structure. The NG sample exhibited a closer interlayer spacing to graphite by shifting the X-ray diffraction (XRD) peak to 26.4°. Due to the defects or strains, both RGO and NG showed larger full width half-maximum. From Raman spectra (Fig. 2) and the the calculation of D/G intensity (*I*_*D*_*/I*_*G*_) listed in Table S2, the *I*_*D*_*/I*_*G*_ ratio of TPRGO slightly increased upon annealing temperature. Although sp^2^ domains are partially restored, the removal of oxygen led to more defects on the structure, inducing smaller and dispersed sp^2^ domains^26^. By introducing nitrogen heteroatom into graphene structure, the *I*_*D*_*/I*_*G*_ ratio reached up to 1.03, indicating more defects in NG.

As shown in Figure S1, the X-ray photoelectron spectroscopy (XPS) curve fitting was performed using a Gaussian-Lorentzian peak shape after subtracting a Shirley background. The sp^2^ peak of the C1s envelope is centered at 284.8 eV. In the RGO sample, the other four components at 285.9, 286.6, 287.5, 288.9 eV are assigned to hydroxyl/phenolic group (C-OH), epoxide group (C-O-C), carbonyl group (>C = O) and carboxyl group (HO-C = O) respectively^27^. Thermal treatment of RGO at high temperature was found to be efficient in the removal of oxygen functional groups, resulting in high C/O ratio. Figure S2 displays the bonding configuration of N atom in NG. The full range analysis (Figure S2a) confirmed the successful doping of nitrogen atoms into the graphene network with 6.79% N atomic content. As shown in Figure S2b, the N1s peaks can be fitted into three ones located at 397.7, 400.5, 402.0 eV, which are related to pyridinic N, pyrrolic N and quaternary N^28^ respectively. The estimated contents of three types of N species were 38%, 38% and 24% respectively, showing that pyridinic and pyrrolic N species were predominant in the NG sample.

### Effect of RGO on nitrobenzene reduction by anaerobic sludge

**Figure 3:**
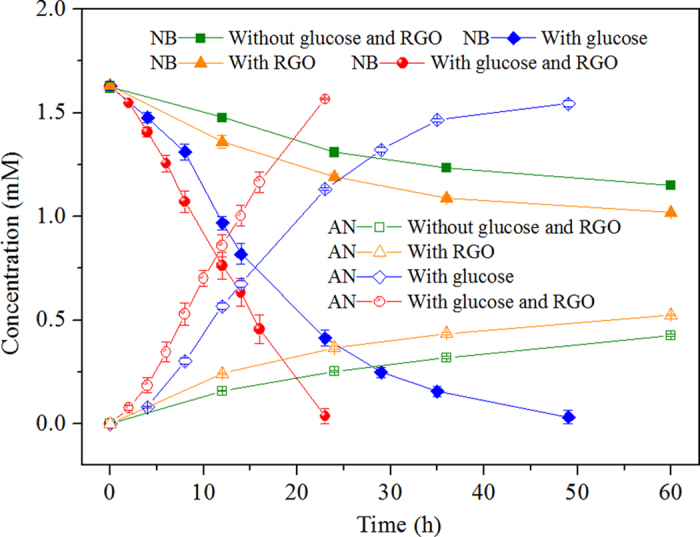
Effect of RGO on nitrobenzene (NB) removal and aniline (AN) formation by anaerobic sludge with glucose as an electron donor (1.6 mM nitrobenzene, 0.55 g VSS L^−1^, pH 7.2, 35 ^°^C, 1 g L^−1^ glucose, 300 mg L^−1^ RGO).

Without the addition of anaerobic sludge, the removal of nitrobenzene was very slow, and aniline could not be detected within 35 h (data not shown). As shown in Fig. 3, without the addition of glucose, nitrobenzene was also slowly transformed by anaerobic sludge within 60 h in the absence and presence of RGO. The measured average reduction rate of nitrobenzene (*V*_NB_) was 61.14 ± 0.48 μmol h^−1^ g-VSS^−1^ in the presence of sludge and glucose without RGO. RGO accelerated the conversion rate of nitrobenzene to aniline and cut the nitrobenzene disappearance time in half, where the *V*_NB_ value calculated from the corresponding RGO-containing system was 124.49 ± 2.91 μmol h^−1^ g-VSS^−1^. Moreover, the good mass balance achieved by summing the nitrobenzene and aniline concentrations (>95%) indicates that aniline was the main product of nitrobenzene reduction in the presence of RGO.

Three fermented intermediates, including acetate, propionate and butyrate, were selected as electron donors. As shown in Figure S3, nitrobenzene was slowly reduced by sludge without additional electron donors, and approximately 65.6% nitrobenzene disappeared within 400 h. In the absence of RGO, none of these volatile fatty acids (VFAs) was efficiently utilized as a carbon source to promote nitrobenzene reduction. Even in the presence of RGO, the nitroreductive rate in each VFA system did not show a significant improvement compared to the experiment without VFA addition, and the concentrations of acetate, propionate and butyrate varied slightly (Figure S4), further supporting the fact that these VFA carbon sources were not metabolized by microbes to reduce nitrobenzene. Similar phenomenon was observed in other studies^29^,^30^,^31^, in which acetate had little impact on the transformation of NACs.

Figure S5 shows the effects of hydrogen and formate on nitrobenzene transformation in the sludge and sludge-RGO systems. With hydrogen, the promotion of nitrobenzene conversion by RGO was insignificant, as seen in Figure S5a. In the presence of formate (Figure S5b), RGO facilitated nitrobenzene disappearance and aniline appearance. It is reasonable to presume that the combined interaction of RGO and sludge-formate was superior to that of RGO and sludge-hydrogen.

### Changing profiles of the microbial community in the presence of RGO

**Figure 4:**
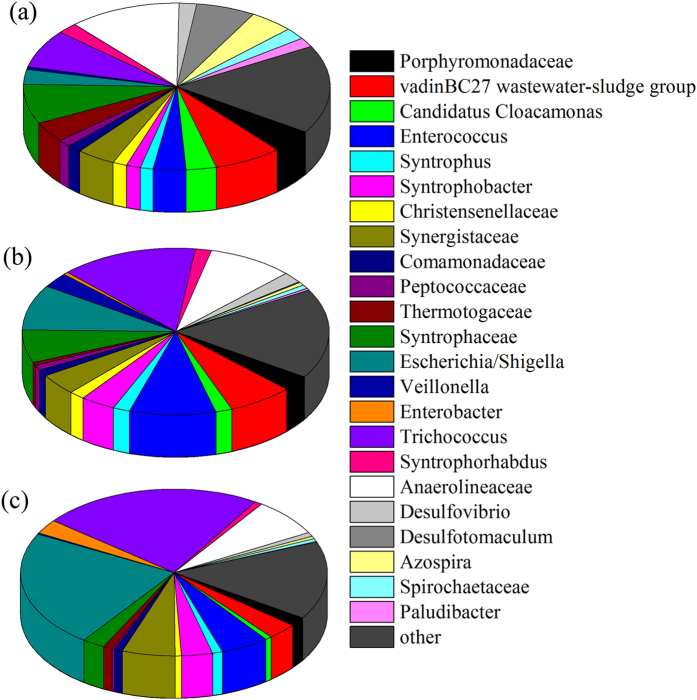
Taxonomic classification of 16 S rRNA gene sequences from bacterial communities of the (**a**) sludge-glucose, (**b**) sludge-glucose-nitrobenzene and (**c**) sludge-glucose-nitrobenzene-RGO systems at the genus level. Relative abundance was calculated as the percentage of the same taxon to the corresponding total sequences for each sample. Genera with less than 1% abundances were summarized as others.

The microbial community diversity in the sludge-glucose, sludge-glucose-nitrobenzene and sludge-glucose-nitrobenzene-RGO systems was analyzed by Illumina sequencing. The number of bacterial sequences in each sample was over 20000, and 1969, 1856 and 1335 operational taxonomic units (OTUs) were detected for the sludge-glucose, nitrobenzene-containing and nitrobenzene-RGO-containing tests, respectively. A total of 247 genera were classified, and 23 genera with a relative abundance ≥1% were selected for further investigation (Fig. 4). In the sludge-glucose system without addition of nitrobenzene and RGO, the bacterial community composition were *Anaerolineaceae* (11.82%), *Syntrophaceae* (7.67%), *Trichococcus* (7.45%), *vadinBC27 wastewater-sludge group* (7.21%), *Desulfotomaculum* (6.24%) and 18 other genera (less than 5% relative abundance). In the sludge-glucose-nitrobenzene system, the enriched bacteria were *Trichococcus* (14.52%), *Enterococcus* (9.11%), *Anaerolineaceae* (9%), *Escherichia* (8.81%), *vadinBC27 wastewater-sludge group* (6.88%), *Syntrophaceae* (6.35%). The dominant genera in the RGO-containing system were *Trichococcus* (23.24%), *Escherichia* (22.66%), *Anaerolineaceae* (7.36%), *Synergistaceae* (5.58%) and *Enterococcus* (5.11%). The potential function of these genera, as summarized in the figure, was also investigated. Four dominant genera (*Escherichia, Enterococcus, Desulfovibrio* and *Enterobacter*) can reduce nitroaromatics to the corresponding aromatic amines^5^,^32^. Among the mentioned genera, *Escherichia, Desulfovibrio* and *Enterobacter* were identified to have electrochemical activity^33^,^34^. At the same time, other electrochemically active bacteria (*Desulfobulbus* and *Geobacter*) also appeared, but at lower abundance. In addition, other dominant genera (such as *Escherichia, Trichococcus, Anaerolineaceae, Syntrophaceae and Synergistaceae*) played the roles of saccharolytic fermentation, consumption and generation of anaerobic intermediates.

RGO is a double-edged sword that can have either a positive or negative influence on the different microorganisms in this study. For instance, in the nitrobenzene-containing and nitrobenzene-RGO-containing tests, the ratio of *Escherichia* and *Enterobacter* increased from 8.81% and 0.54% to 22.66% and 2.77% after adding RGO. In contrast, the proportion of *Desulfovibrio*, *Geobacter* and *Desulfobulbus* decreased from 2.07%, 0.46% and 0.22% to 0.79%, 0.13% and 0.18%, respectively. The variation of the electrochemically active bacteria might affect the electron transfer in the anaerobic system.

### Enhancement mechanisms of biological nitrobenzene reduction by RGO

The transfer of reducing equivalents to the nitro group is required in the anaerobic degradation of NACs. The addition of an electron donor is necessary to support the reduction processes^30^, such as glucose, hydrogen, formate, or starch. In general, intracellular electron transfer to nitroreductases is indispensable in nitrobenzene bio-reduction. Yan *et al.* recently reported that carbon nanotubes could enhance nitrobenzene reduction by altering the electron flow route^35^. Extracellular electron transfer to nitrobenzene via graphene is another promising pathway for nitrobenzene degradation. Meanwhile, a number of previous studies reported that carbon-based materials exhibited high electrical conductivity and facilitated electron transfer^15^,^36^. They could act as electron conductors or redox mediators, which resulted in the enhancement of electron transfer.

**Figure 5:**
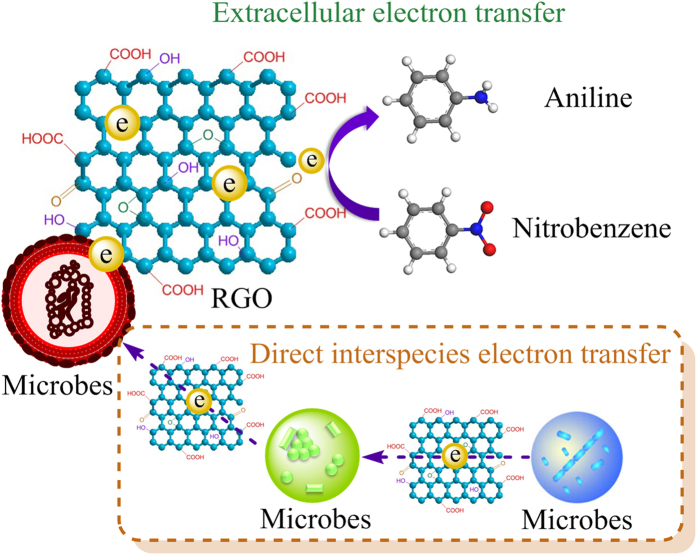
Schematic diagram of RGO involved in extracellular electron transfer during nitrobenzene transformation by mixed anaerobic culture.

Accordingly, RGO is supposed to accelerate extracellular electron transfer, which further mediated nitrobenzene reduction (Fig. 5). Electrochemically active bacteria, such as *Escherichia, Desulfovibrio* and *Enterobacter*, had been detected in both sludge and sludge-RGO systems. RGO could contact with these bacteria and affect its proportion, as evidenced by scanning electron microscope (SEM) and high-throughput sequencing analysis. From the SEM images (Figure S6a), there were mainly two types of cell morphology (plate and rod shapes) in the RGO-free reactor. Cells appeared to contact with RGO (such as red boxes in Figure S6b) in the RGO-adding reactor and made it possible to transfer electrons from microbes to RGO and to bridge the connections of different cells, which is similar to the interaction between nanoFe_3_O_4_ particles and microbes^37^. Simultaneously, RGO can absorb nitrobenzene and form π-π interactions with aromatic rings, which resulted in the transfer of electrons to nitrobenzene.

Additionally, RGO could mediate direct interspecies electron transfer (DIET) and activate nitrobenzene molecules. In recent years, it was suggested that DIET is an alternative mechanism for electron exchange through biological electrical connections^38^, in contrast to interspecies hydrogen/formate electron transfer. Liu *et al.* reported that activated carbon promoted DIET^39^. RGO was also expected to accelerate DIET between microbes, which might be favorable for nitroreductase to accept electrons. Here, RGO plays the role of “extended nanowire”. The activation of nitrobenzene by graphene has been proposed using density functional theory calculations, and the unsaturated carbon atoms at the edges of graphene and defects on graphene might possess catalytic activity^11^.

### Role of oxygen moieties on the RGO surface in nitrobenzene reduction

**Figure 6:**
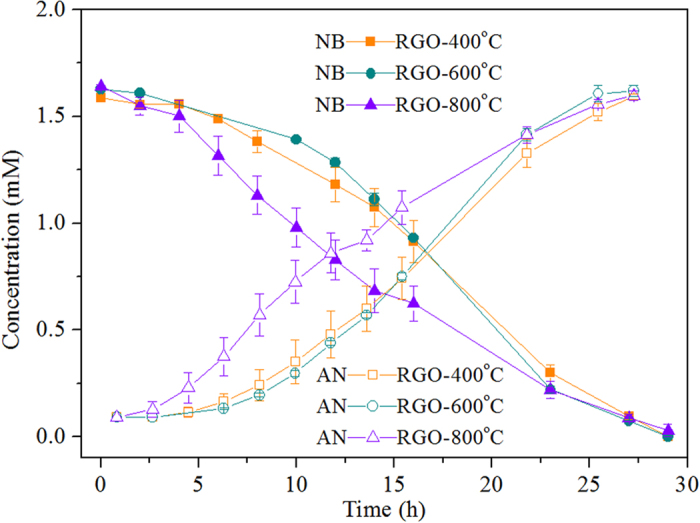
Comparison of thermally pretreated RGO (TPRGO) on nitrobenzene reduction and aniline formation by anaerobic sludge (1.6 mM nitrobenzene, 1 g L^−1^ glucose, 0.55 g VSS L^−1^, pH 7.2, 35 ^°^C, 300 mg L^−1^ TPRGO).

As shown in Fig. 6, the *V*_NB_ values of RGO-400 °C, RGO-600 °C and RGO-800 °C were 99.44 ± 0.95, 103.60 ± 0.25, and 99.94 ± 2.26 μmol h^−1^ g-VSS^−1^, respectively. The TPRGO promoted nitrobenzene transformation compared to the control without RGO, but the reduction rate, in spite of the annealing temperature, was lower than in the presence of RGO without pretreatment, i.e., 124.49 ± 2.91 μmol h^−1^ g-VSS^−1^. As evidenced from the C1s spectrum determined by XPS (Figure S1), RGO without pretreatment was rich in oxygen species, while thermal modification could result in the decline of carbonyl groups. According to literature^40^, the removal of carbonyl group implied the decrease of quinone groups on the surface of carbon materials, which have been proposed to act as redox mediators to enhance extracellular electron transfer^41^,^42^. Therefore, compared to untreated RGO, the slower nitrobenzene removal with anaerobic sludge was likely due to the reduction of quinone groups on the surface of RGO through thermal pretreatment. In addition to quinone groups served as electron shuttling moieties, RGO could also catch electrons and achieve electron transfer through the graphitic matrix. It was reported that the electrical conductivity of graphene could be increased during thermal annealing^43^, which was able to accelerate electron transfer. The results from this study indicate that the negative effect of oxygen moieties reduction on the RGO would be more powerful on biological nitrobenzene reduction, compared to the positive function of the electrical conductivity increase of RGO upon annealing. As a consequence, oxygen moieties, e.g., quinone groups, were of great significance in the electron transfer occurring on RGO for biological nitrobenzene removal with anaerobic sludge.

### Nitrobenzene reduction by nitrogen-doped graphene

**Figure 7:**
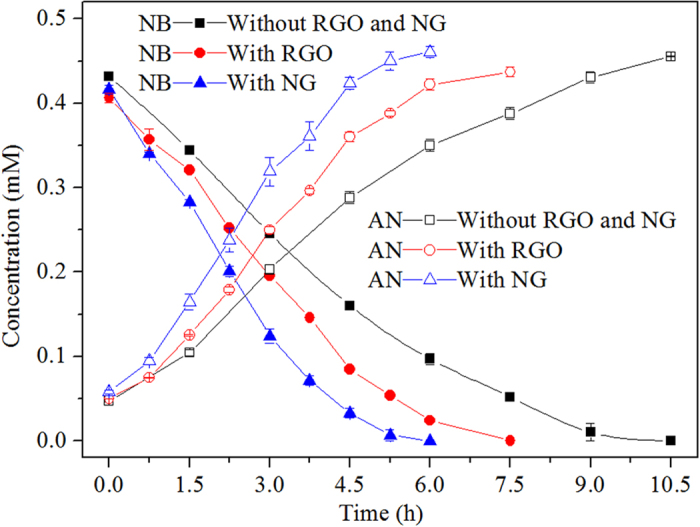
Evaluation of NG and RGO on nitrobenzene conversion (0.4 mM nitrobenzene, 1 g L^−1^ glucose, 0.55 g VSS L^−1^, pH 7.2, 35 ^°^C, 300 mg L^−1^ RGO or NG).

The contribution of NG to nitrobenzene reduction by mixed culture was also investigated compared to RGO (Fig. 7). RGO increased the *V*_NB_ from 84.25 ± 2.88 μmol h^−1^ g-VSS^−1^ (absence of RGO) to 114.57 ± 1.65 μmol h^−1^ g-VSS^−1^. Furthermore, in the NG system, the *V*_NB_ value was enhanced to 140.31 ± 3.97 μmol h^−1^ g-VSS^−1^. NG increased the nitrobenzene conversion rate by 66.5% and 22.5%, respectively, compared to the control (absence of RGO and NG) and RGO-containing systems. The oxygen content in NG was estimated to be 4.05% from XPS results, which was similar to that in RGO-800 °C. However, the content of nitrogen dopant atoms significantly altered, as evidenced from XPS analysis, which could influence the property of graphene such as electrical conductivity and catalytic activity.

The better performance of converting nitroaromatics by sludge-NG compared to sludge-RGO was likely due to the enhanced electron transfer and catalytic properties. Firstly, the Freundlich model was adopted to simulate the nitrobenzene adsorption behavior on RGO and NG:


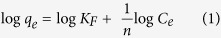


where *q*_*e*_ is the adsorbed nitrobenzene per gram of RGO/NG (mmol g^−1^), *C*_*e*_ is the equilibrium concentration of nitrobenzene in solution (mmol L^−1^), *K*_*F*_ and n are the adsorption constant and the Freundlich linearity index.

As presented in Table S3, the Freundlich model fitted the data well and the *K*_*F*_ values of RGO and NG were 1.426 and 1.428, respectively, indicating insignificant difference of nitrobenzene adsorption capability between RGO and NG. Secondly, in order to investigate the electron transfer capability of RGO and NG, both RGO- and NG-modified GC electrodes were fabricated and compared in a three-electrode cell with 5.0 mmol L^−1^ K_3_[Fe(CN)_6_]/K_4_[Fe(CN)_6_] and 0.1 mol L^−1^ KCl solution. As shown in Figure S7, a pair of reversible redox peaks appeared in the cyclic voltammogram. Compared with RGO-modified electrode, the peak current of NG-modified electrode was increased by one-fold, demonstrating that the electron transfer ability of NG was superior to the RGO. N^+^ ion implantation similarly increased the electrical conductivity of carbon paper and enhanced microbial adhesion on its surface^44^. Accordingly, the electron transferred to the adsorbed nitrobenzene on NG surface was anticipated to be faster than that on RGO framework. Thirdly, the impact of nitrogen doping on the N-O bond lengths of the nitro group has also been calculated using density functional theory (DFT), as shown in Figure S8. The N-O bond length of free nitrobenzene is 1.256 Å and it changed to 1.550, 1.260, 1.260 Å after adsorbed with pyrrolic N, pyridinic N and quaternary N in the NG network. Overall, nitrogen, particularly pyrrolic N, could activate the N-O bond, which was favorable for the reduction of nitrobenzene molecule. As a summary, in comparison to RGO, although nitrobenzene adsorption on the NG was not significantly increased, the dopant of nitrogen in the graphene could not only activate the N-O bond of adsorbed nitrobenzene molecule but also accelerate the electron transfer on its surface. And surface modification of graphene would remarkably affect its capacity of extracellular electron transfer, which further could regulate the anaerobic transformation of contaminants.

In conclusion, the present study demonstrates that RGO could be involved in the extracellular electron transfer of microorganisms to facilitate anaerobic nitrobenzene removal with mixed cultures. The removal of oxygen moieties from RGO surface, such as quinone groups, resulted in the decreased biotransformation rate of nitrobenzene, while the surface modification by doping nitrogen into graphene network promoted biological nitrobenzene conversion. Therefore, graphene-based carbon nanomaterials and their functional derivatives have great potentials to accelerate the biological redox reactions of various environmental contaminants via extracellular electron transfer. Furthermore, coupling of functional nanomaterials with microorganisms may provide a new opportunity of developing advanced wastewater treatment and environmental remediation technologies.

## Methods

### Materials

Nitrobenzene (≥99.0%, Sigma Aldrich, USA) and aniline (≥99.5%, Sinopharm Chemical Reagent Ltd., China) were used as received. RGO and NG were purchased from Chengdu Organic Chemicals (Sichuan, China). RGO was chemically reduced from graphene oxide, and NG was a product of the reaction of chemically reduced graphene oxide with nitrogen-containing polymers at temperature above 900 °C. Mixed anaerobic microorganisms were taken from a full-scale, expanded sludge-bed reactor treating starch wastewater in Shandong Province, China. Before experiments, the anaerobic sludge was cultivated at a nitrobenzene concentration of 1.6 mM for at least five cycles. A mixture of glucose and acetate at the ratio of 4:1 was utilized as electron donors and each cycle was operated at an organic loading of 0.32 kg COD m^−3^ d^−1^ for four days.

### TPRGO

The TPRGO was prepared according to a previous study^45^. Briefly, RGO was placed in a tube furnace initially at room temperature and was heated to 400 °C, 600 °C, or 800 °C in flowing argon. The final heating temperature was maintained for half an hour. After cooling to room temperature, the treated RGO was immediately preserved in an anaerobic chamber (Bactron, USA). The TPRGO at various temperatures was labeled as RGO-400 °C, RGO-600 °C and RGO-800 °C, respectively.

### Characterization of RGO, TPRGO and NG

Transmission electron microscopy (TEM) images of materials were observed from a JEOL-2010 high-resolution transmission electron microscope (JEOL Ltd., Japan). XRD tests were performed on Rigaku TTR-III with Cu Kα radiation (Rigaku Corp., Japan). Raman spectra were collected on LABRAM-HR Raman spectrometer (JY Co., France) with an excitation wavelength of 514.5 nm generated by an Agon laser. XPS was determined by using ESCALAB 250 with a monochromatic Al Kα X-ray source (Thermo-VG Scientific Inc., USA).

### Batch tests

All batch reactions were conducted in 180 mL serum bottles containing 100 mL synthetic basal medium (per liter), 3.5 g NaHCO_3_, 0.4 g NH_4_Cl, 0.08 g Na_2_SO_4_, 0.072 g MgCl_2_·6 H_2_O, 0.01 g CaCl_2_·2 H_2_O, 0.052 g KCl, 0.5 g L-Cysteine monohydrochloride, 1 g resazurin and 1 mL of trace element mixture. The trace element mixture was prepared by mixing 0.2 g H_3_BO_3_, 0.68 g MnSO_4_·4 H_2_O, 1.2 g CoCl_2_·6 H_2_O, 1.1 g CuCl_2_·2 H_2_O, 0.1 g Na_2_MoO_4_·2 H_2_O, 3.2 g ZnSO_4_·7 H_2_O, 3.2 g FeSO_4_·7 H_2_O, 0.5 g NiCl_2_·6 H_2_O, and 0.6 g EDTA in one liter of deionized water. The sludge concentration was maintained at 550 mg volatile suspended solids (VSS) per liter in all experiments. The initial nitrobenzene concentration ranged from 0.4 to 1.6 mM. The concentration of RGO, NG and TPRGO, if added, was set at 300 mg L^−1^. The serum bottles were sealed with butyl rubber stoppers, flushed with nitrogen for 15 mins to purge oxygen and then incubated at 35 °C with shaking at 200 rpm. The initial pH of the solution was adjusted to 7.2 with HCl solution.

Four batch tests were set up, as summarized in Table 1. Batch test 1 was conducted to determine the effect of RGO on nitrobenzene reduction using glucose as an electron donor. To probe the difference of various electron donors for nitrobenzene reduction in the presence of RGO, three VFAs, hydrogen and formate were introduced in batch test 2. The VFAs included acetate, propionate and butyrate, and their concentrations were equal to the COD of glucose. The headspace of the bottle included 20% hydrogen and 80% nitrogen gas in the sludge-hydrogen system, and the initial formate concentration was set to 10 mM in the sludge-formate system. Batch test 3 was conducted to determine the influence of oxygen moieties on the conversion rates of nitrobenzene by introducing TPRGO. Finally, the effect of nitrogen doping on nitrobenzene reduction was evaluated in the presence of glucose in batch test 4. Each experiment was repeated at least twice.

### Analysis

At appropriate time intervals, samples were taken and immediately mixed with methanol (1:9, v:v) to extract nitrobenzene and its reductive intermediates that might be adsorbed by RGO, TPRGO or NG. After filtering through a 0.22 μm membrane, the dilutions were analyzed by high-performance liquid chromatography (HPLC) as soon as possible. HPLC analysis was performed on an Agilent 1260 equipped with a diode array detector and a 4.6 × 250 mm Eclipse Plus C18 column at 30 °C (Agilent Technologies, USA). The mobile phase was methanol:water (1:1) with a flow rate of 1.0 mL min^−1^, and the detection wavelength was 254 nm. VFAs were acidified with formic acid and were determined by a 7890 gas chromatograph (Agilent Technologies, USA). The total suspended solids and VSS were analyzed following the standard methods^46^. The average reduction rate of nitrobenzene (*V*_NB_, μmol h^−1^ g-VSS^−1^) was calculated by dividing the difference between the initial and finial nitrobenzene concentration by the reaction time and sludge concentration. In the adsorption test, the initial concentrations of nitrobenzene ranged between 0.16 to 1.6 mM in the presence of 300 mg L^−1^ RGO or NG at 30 °C. The working electrodes including RGO- and NG-modified GC ones were fabricated according to a previous study^47^. A three-electrode cell, including working electrode, Ag/AgCl reference electrode and platinum wire counter electrode, was used for electrochemical measurement in a mixed solution with 5.0 mmol L^−1^ K_3_[Fe(CN)_6_]/K_4_[Fe(CN)_6_] and 0.1 mol L^−1^ KCl at 100 mV S^−1^ scan rate. The details for DFT calculations are provided in the Supporting Information (SI).

The samples for the analysis of morphology and microbial community diversity were collected after 17–18 days reaction, which was based on the reaction time for nitrobenzene reduction in absence of additional electron donors. After centrifugation, the pellets were fixed with 5% (wt/vol) glutaraldehyde and were dehydrated using a graded series of ethanol solutions. Then, the morphology of the samples was characterized by SEM (Supra 40, Zeiss Co., Germany). The total DNA was extracted using the PowerSoilTM DNA isolation kit (Mo-Bio Laboratories Inc., USA). The samples were analyzed by high-throughput sequencing on an Illumina platform (Illumina Miseq). The variable region V3-V4 of the 16S rRNA gene was amplified with a bacteria primer set of 341F (5′-CCTACGGGNGGCWGCAG-3′) and 805R (5′-GACTACHVGGGTATCTAATCC-3′) using a PCR instrument (T100TM Thermal Cycler). The primers of the 16S V3-V4 region for different samples were encoded with different barcodes and were combined into a single library for sequencing on the Miseq system. The 16S rRNA gene sequences were classified into OTUs with 97% similarity.

## Additional Information

**How to cite this article**: Li, L. *et al.* Facilitated biological reduction of nitroaromatic compounds by reduced graphene oxide and the role of its surface characteristics. *Sci. Rep.* **6**, 30082; doi: 10.1038/srep30082 (2016).

## Supplementary Material

Supplementary Information

## Figures and Tables

**Table 1:** Summary of experimental setup for each batch test.

Batch test	Goal	Electron donor	RGO surface modification	Description
1	Effect of RGO on nitroreduction using glucose as an electron donor	Glucose	No	Interaction of RGO with sludge-glucose
2	Effect of RGO on nitroreduction using three VFAs or two electron carriers as electron donors	Acetate, propionate, butyrate, hydrogen, formate	No	Interaction of RGO with sludge-three different VFAs (acetate, propionate and butyrate) or sludge-two electron carriers (hydrogen and formate)
3	Influence of oxygen moieties on nitrobenzene transformation	Glucose	Yes	Removal of functional groups on RGO by thermal annealing
4	Impact of nitrogen doping into graphene network on nitroaromatic conversion	Glucose	Yes	Surface modification of graphene by nitrogen doping
