# Supplementary material for: Facilitated biological reduction of nitroaromatic compounds by reduced graphene oxide and the role of its surface characteristics
Source: Sci Rep. 2016 Jul 21;6:30082. doi: 10.1038/srep30082 (PMC4954959; doi:10.1038/srep30082)
Supplement: Supplementary Information [file srep30082-s1.doc]

**Supplementary Information**

**Facilitated** **biological reduction of nitroaromatic compounds by reduced graphene oxide and the role of its surface characteristics**

Lei Li^a^, Qi Liu^a^, Yi-Xuan Wang^a^, Han-Qing Zhao^a^, Chuan-Shu He^a^, Hou-Yun Yang^a^, Li Gong^a^, Yang Mu^a,b^*, Han-Qing Yu^a^

^a^CAS Key Laboratory of Urban Pollutant Conversion, Collaborative Innovation Centre of Suzhou Nano Science and Technology, Department of Chemistry, University of Science and Technology of China, Hefei, China

^b^Jiangsu Key Laboratory of Chemical Pollution Control and Resources Reuse, School of Environmental and Biological Engineering, Nanjing University of Science and Technology, Nanjing 210094, Jiangsu Province, China

***Corresponding author:**

Prof. Yang Mu, Fax: +86 551 63607907; E-mail address: [yangmu@ustc.edu.cn](mailto:yangmu@ustc.edu.cn)

**Methods**

**Density functional theory (DFT) calculations**

The activation of the N-O bond of the nitrobenzene molecular is the essential step in the nitrobenzene hydrogenation process^1^. DFT calculations with and without nitrogen-doped graphene (NG) geometry optimization were carried out to explore the contribution of nitrogen to the activation of the nitrobenzene molecular. Our DFT calculations were performed employing the ultrasoft pseudopotential for the ion-electron interactions and generalized gradient approximation (GGA)^2^ with the Predew-Burke-Ernzerhof (PBE)^3^ functionals for the exchange-correlation energy of electrons in the supercell approach as implemented in CASTEP program^4^. The geometry optimization convergence thresholds were set to be medium based on the following points: (1) an energy tolerance of 2 × 10^−5^ eV per atom; (2) a maximum force tolerance of 0.05 eV/Å; and (3) a maximum displacement tolerance of 2 × 10^−3^ Å. As shown in Figure S9, the NG was model as an (8×8) single-layer graphene doped with pyrrolic, pyridinic and quaternary N atoms according to the XPS results (Figure S2b). The periodic boundary conditions were imposed on the model in a supercell with a large vacuum region of 15 Å along the c axis to avoid the unwanted interaction between its period images.

**References**

1. Gao, Y. J., Ma, D., Wang, C. L., Guan, J. & Bao, X. H. Reduced graphene oxide as a catalyst for hydrogenation of nitrobenzene at room temperature. *Chem. Commun.* **47**, 2432-2434 (2011).
2. Vanderbilt, D. Soft Self-Consistent Pseudopotentials in a Generalized Eigenvalue Formalism. *Phys. Rev. B* **41**, 7892-7895 (1990).
3. Perdew, J. P., Burke, K. & Ernzerhof, M. Generalized gradient approximation made simple. *Phys. Rev. Lett.* **77**, 3865-3868 (1996).
4. Segall, M. D. et al. First-principles simulation: ideas, illustrations and the CASTEP code. *J. Phys.-Condens. Mat.* **14**, 2717-2744 (2002).
5. Pereira, R. A., Pereira, M. F. R., Alves, M. M. & Pereira, L. Carbon based materials as novel redox mediators for dye wastewater biodegradation. *Appl. Catal. B-Environ.* **144**, 713-720 (2014).
6. Amezquita-Garcia, H. J., Razo-Flores, E., Cervantes, F. J. & Rangel-Mendez, J. R. Activated carbon fibers as redox mediators for the increased reduction of nitroaromatics. *Carbon* **55**, 276-284 (2013).
7. Toro, E. E. R. D., Celis, L. B., Cervantes, F. J. & Rangel-Mendez, J. R. Enhanced microbial decolorization of methyl red with oxidized carbon fiber as redox mediator. *J. Hazard. Mater.* **260**, 967-974 (2013).
8. Pereira, L. et al. Thermal modification of activated carbon surface chemistry improves its capacity as redox mediator for azo dye reduction. *J. Hazard. Mater.* **183**, 931-939 (2010).
9. Wang, J., Wang, D., Liu, G. F., Jin, R. F. & Lu, H. Enhanced nitrobenzene biotransformation by graphene-anaerobic sludge composite. *J. Chem. Technol. Biot.* **89**, 750-755 (2014).
10. Colunga, A., Rangel-Mendez, J. R., Celis, L. B. & Cervantes, F. J. Graphene oxide as electron shuttle for increased redox conversion of contaminants under methanogenic and sulfate-reducing conditions. *Bioresource Technol.* **175**, 309-314 (2015).
11. Toral-Sánchez, E., Valdés, J. A. A., Aguilar, C. N., Cervantes, F. J. & Rangel-Mendez, J. R. Role of the intrinsic properties of partially reduced graphene oxides on the chemical transformation of iopromide. *Carbon* **99**, 456-465 (2016).

**Table S1. Literature comparison on reductive transformation of recalcitrant pollutants with addition of carbon materials**

| Literature | Carbon material/ Surface modification | Chemical/ biological reduction | Main objects | Main results |
| --- | --- | --- | --- | --- |
| Carbon based materials as novel redox mediators for dye wastewater biodegradation^5^ | Activated carbon (AC_H2_), mesoporous carbons (CXA, CXB) and carbon nanotubes (CNT);  No surface modification | Biological reduction | How carbon materials affect the biological treatment of textile wastewater | 1. All the carbon materials could achieve ~85% of Mordant Yellow 10 and 70% of Reactive Red 120 colour removal. 2. CXB and CNT removed 98% colour of Acid Orange 10 which was unbiodegradable in the absence of carbon materials. 3. For the biotransformation of Mordant Yellow 10 and Red 120, the rates increased as follows: control < AC_H2_< CXA < CXB < CNT. |
| Activated carbon fibers as redox mediators for the increased reduction of nitroaromatics^6^ | Activated carbon fibers (ACFs);  Oxidation and thermal modification | Chemical reduction | The effect of ACFs and its chemical properties on the nitroaromatic transformation | 1. ACFs could act as redox mediators to transform the nitroaromatic compounds. 2. The oxidation and thermal modification changed the redox properties and the redox mediating capacity of the oxidated ACFs was enhanced due to the increased quinone groups (1.68 times). |
| Enhanced microbial decolorization of methyl red with oxidized carbon fiber as redox mediator^7^ | Activated carbon fiber (ACF);  Chemical oxidation | Biological reduction | The use of ACF as a redox mediator for the anaerobic conversion of the azo dye | 1. ACF could accelerate the biodegradation of methyl red. 2. Chemical modification of ACF with HNO_3_ increased its redox-mediating capacity in biological assays. 3. The biofilm formed on ACF decreased the reductive transformation of methyl red and the electron transport from ACF to dye was the rate-determining step. |
| Thermal modification of activated carbon surface chemistry improves its capacity as redox mediator for azo dye reduction^8^ | Activated carbon (AC);  Chemical oxidation and thermal modification | Both | The effect of thermal modification of activated carbon on azo dye degradation | 1. Compared with control, AC showed a 9-fold increase in the reduction of dyes. 2. The reductive rate generally followed the trend: AC_HNO3_ < AC_O2_ < AC_0_ < AC_N2_ < AC_H2_. 3. In biotic system, AC_H2_ increased 1- and 4.5- fold the decolourization rates of Mordant Yellow 10 and Reactive Red 2. |
| Enhanced nitrobenzene biotransformation by graphene-anaerobic sludge composite^9^ | Reduced graphene oxide (RGO);  No surface modification | Biological reduction | The function of RGO in the nitrobenzene bioreduction by anaerobic sludge | 1. RGO increased the dehydrogenase activity of anaerobic sludge by 2-fold and redox active species appeared in the supernatant from RGO system. 2. Bound and free extracellular polymeric substances contributed to the direct biological degradation of nitrobenzene and bound EPS might interact with secreted redox active species. |
| Graphene oxide as electron shuttle for increased redox conversion of contaminants under methanogenic and sulfate-reducing conditions^10^ | Graphene oxide (GO);  No surface modification | Both | The role of GO in the redox conversion of nitroaromatics and azo dyes | 1. In abiotic reduction, GO increased the transformation of reactive red and 3-chloronitrobenzene by 10- and 7.6-fold. 2. In biotic reduction, the conversion of reactive red was also enhanced by 2- and 3.6-fold. 3. The promoted reduction might be related to the proper size distribution and redox potential of GO. |
| Role of the intrinsic properties of partially reduced graphene oxides on the chemical transformation of iopromide^11^ | Graphene oxide (GO)/ reduced graphene oxide (rGO);  Thermal modification | Chemical reduction | The impact of GO, rGO and its oxygen functional groups on the conversion of iopromide | 1. After the removal of different oxygenated groups from GO, the amorphous structure changed to a more crystalline one. 2. Reduced graphene oxide promoted a greater extent of iopromide transformation up to 5.2-fold. 3. The catalytic activity was correlated to the reduction degree of GO. |
| Present work | Reduced graphene oxide (RGO);  Thermal modification and nitrogen doping | Biological reduction | How RGO participate in the biotransformation of nitroaromatics and the influence of RGO surface characteristics | 1. RGO could be involved in the extracellular electron transfer of microorganisms to facilitate anaerobic nitrobenzene removal with mixed cultures. 2. The removal of oxygen moieties from RGO surface resulted in the decreased rate of nitroreduction. 3. The surface modification by doping nitrogen into graphene network promoted nitrobenzene conversion. |

**Table S2. Peaks of D band and G band and *I_D_/I_G_*ratio of RGO, TPRGO and NG**

| Material | D band (cm^-1^) | G band (cm^-1^) | *I_D_/I_G_* ratio |
| --- | --- | --- | --- |
| RGO | ~1353 | ~1586 | 0.88 |
| RGO-400^o^C | ~1344 | ~1588 | 0.87 |
| RGO-600^o^C | ~1343 | ~1595 | 0.89 |
| RGO-800^o^C | ~1351 | ~1595 | 0.96 |
| NG | ~1367 | ~1588 | 1.03 |

Note: Raman spectra of all samples exhibited two dominant characteristic peaks: the D band (~1350 cm^-1^) deriving from the doubly resonant disorder-induced mode and the G band (~1590 cm^-1^) resulting from in-plane vibration of sp^2^ carbon atoms.

**Table S3.** **Simulation of nitrobenzene adsorption on RGO and NG using Freundlich adsorption isotherm (0.16-1.6 mM nitrobenzene, 300 mg L^-1^ RGO/NG, 30^o^C)**

| Adsorbent | *K_F_*  mmol g^-1^ (L mmol^-1^)^1/n^ | n | *R^2^* |
| --- | --- | --- | --- |
| RGO | 1.426 | 1.642 | 0.987 |
| NG | 1.428 | 0.955 | 0.993 |

**Figure Captions**

**Figure S1.** C1s XPS spectra of reduced graphene oxide (RGO).

**Figure S2.** (a) XPS measurement of nitrogen-doped graphene (NG), and (b) N1s peak assignment of NG.

**Figure S3.** Effect of various electron donors on nitrobenzene (NB) removal and aniline (AN) formation with anaerobic sludge in the absence (a) and presence (b) of RGO (1.6 mM nitrobenzene, 1.07 g COD L^-1^ VFAs, 0.55 g VSS L^-1^, pH 7.2, 35^o^C, 300 mg L^-1^ RGO).

**Figure S4.** Variation of volatile fatty acids during nitrobenzene removal in the absence and presence of RGO (1.6 mM nitrobenzene, 1.07 g COD L^-1^ VFAs, 0.55 g VSS L^-1^, pH 7.2, 35^o^C, 300 mg L^-1^ RGO).

**Figure S5.** Effect of hydrogen (a) or formate (b) as electron donors on nitrobenzene transformation with anaerobic sludge in the presence of RGO (1.6 mM nitrobenzene, 20% hydrogen or 10 mM formate, 0.55 g VSS L^-1^, pH 7.2, 35^o^C, 300 mg L^-1^ RGO).

**Figure S6.** SEM images of anaerobic sludge: (a) in the absence of RGO, and (b) in the presence of RGO.

**Figure S7**. Cyclic voltammograms of RGO- or NG- modified GC electrode at 100 mV S^-1^ scan rate in the presence of 5.0 mmol L^-1^ K_3_[Fe(CN)_6_]/K_4_[Fe(CN)_6_] and 0.1 mol L^-1^ KCl solution. A three-electrode cell was used in the measurement, RGO- or NG- modified GC as working electrode, an Ag/AgCl reference electrode and a platinum wire counter electrode.

**Figure S8.** The N-O bond length of (a) free nitrobenzene molecule, and optimized structures of nitrobenzene adsorption on the (b) pyrrolic N, (c) pyridinic N and (d) quaternary N sites of the NG (The numbers in the figures have a unit of Å).

**Figure S9.** Optimized structures of NG with pyrrolic, pyridinic and quaternary N atoms.

**Figure S1**

**Figure S2**

(a)

(b)

**Figure S3**

**Figure S4**

(b)

(a)

**Figure S5**


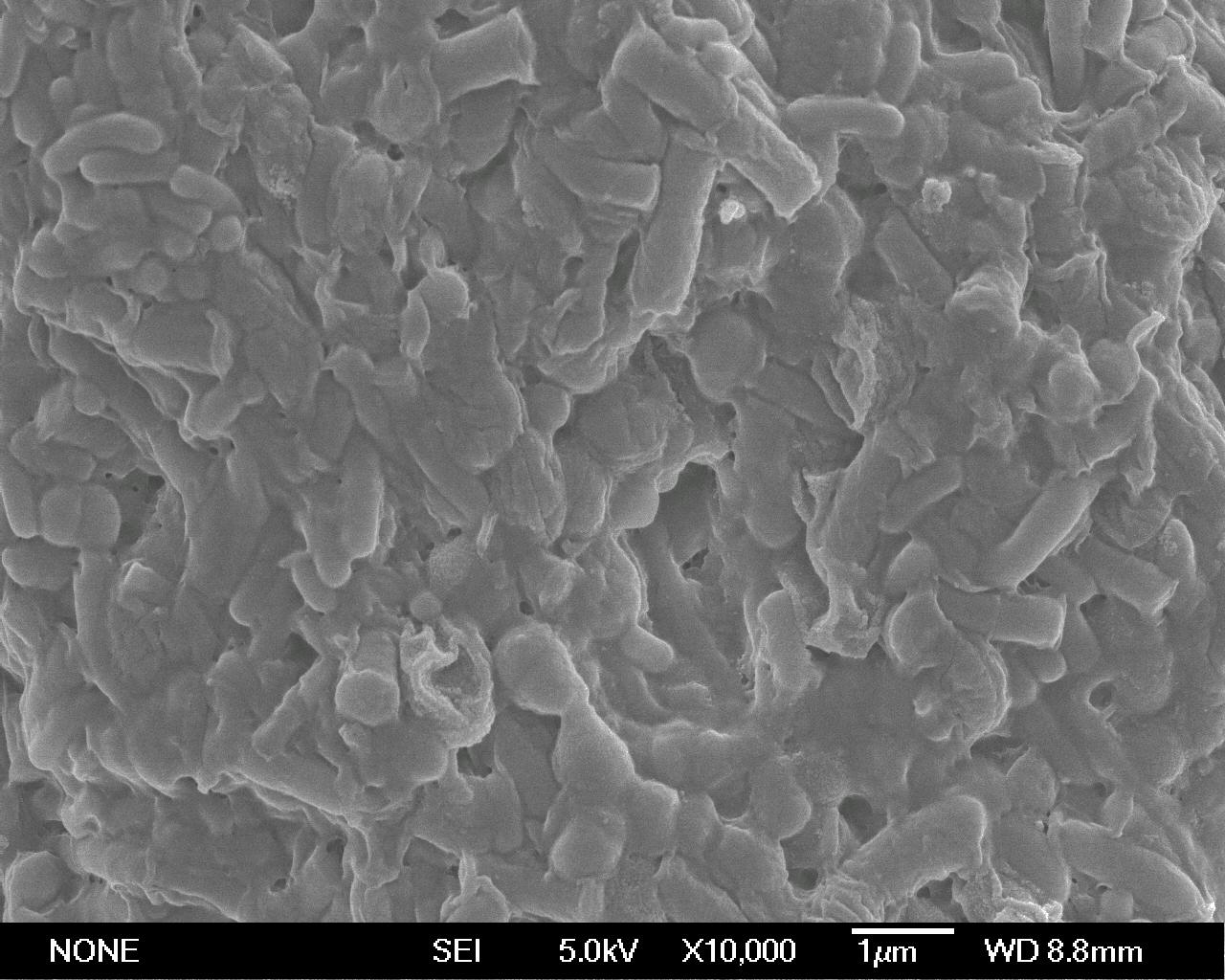

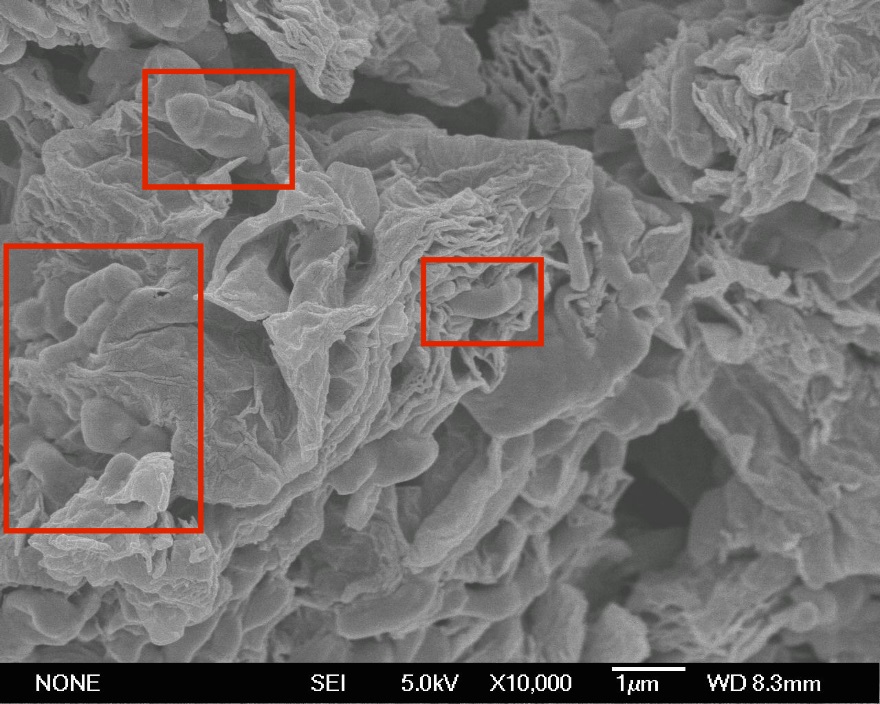


(a)

(b)

**Figure S6**

**Figure S7**

**
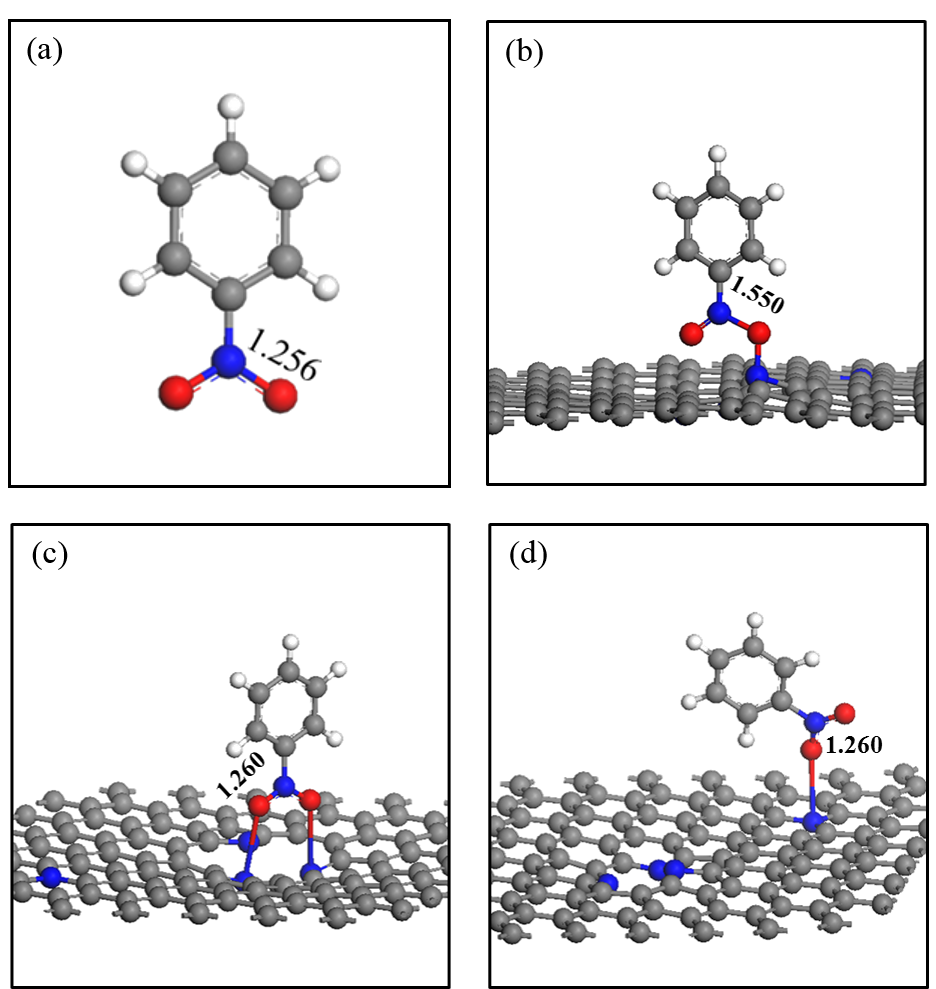
**

**Figure S8**


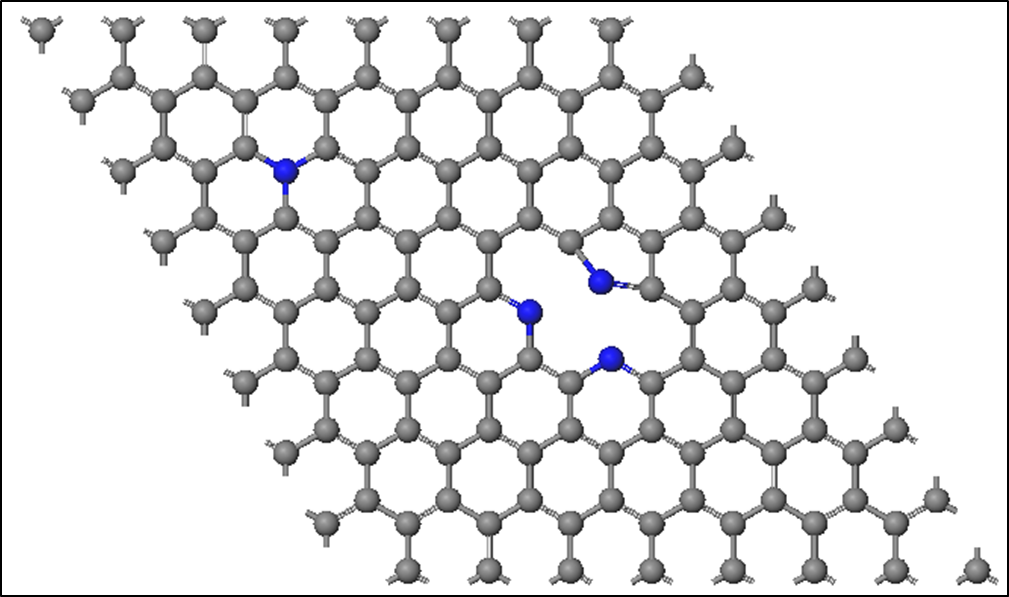


**Figure S9**
